# Editorial: Microbial interactions: from mechanisms to applications in the fight against antimicrobial resistance

**DOI:** 10.3389/fmicb.2026.2003200

**Published:** 2026-09-29

**Authors:** Uelinton M. Pinto, Thiago M. A. dos Santos, Ali Tahrioui

**Affiliations:** 1 Laboratory of Food Microbiology, Food Research Center, Department of Food and Experimental Nutrition, School of Pharmaceutical Sciences, University of São Paulo (USP), São Paulo, Brazil; 2 Department of Chemistry and Chemical Biology, Northeastern University, Boston, MA, United States; 3 Department of Biology, Northeastern University, Boston, MA, United States; 4 Univ Rouen Normandie, Univ Caen Normandie, Normandie Univ, CBSA UR 4312, Rouen, France

**Keywords:** antagonism, antimicrobial resistance, antivirulence, bacteriocins, biofilms, microbial signaling, quorum sensing

## Introduction

Microbial communities are dynamic and interconnected systems in which interactions among microorganisms shape their structure, behavior, survival, and ecological success. These interactions can promote cooperation, and collective resistance, but can also be shaped by competition in the form of antagonism, amensalism, predation and cheating among its members (Faust and Raes, 2012). Understanding the molecular mechanisms and ecological principles underlying these processes (from cooperative interactions such as quorum sensing (QS), adhesion, and cross-feeding to competitive mechanisms involving antagonism and bacteriocin production) is essential for identifying new opportunities to modulate microbial communities and control pathogenicity. This knowledge is relevant in the context of antimicrobial resistance (AMR), where the widespread misuse of antibiotics has accelerated the emergence of multidrug-resistant pathogens while the lack of innovation in the development of new antibiotics has further limited available treatment options. Together, these challenges underscore the need for innovative strategies that target microbial behavior and interactions rather than bacterial viability alone (Santos et al., 2026). Considering the growing threat of AMR, the Research Topic “*Microbial interactions: from mechanisms to applications in the fight against antimicrobial resistance*” brings contributions exploring microbial communication in the form of QS and quorum quenching interactions, biofilm formation and disruption, AMR, stress responses, gene regulation, and antivirulence strategies. Emphasis is placed on understanding intercellular signaling and developing approaches capable of disarming pathogens while minimizing the selective pressure that drives the emergence and dissemination of AMR. Together, the contributions to this Research Topic illustrate the complexity of microbial interactions and highlight how a deeper understanding of these processes can provide both fundamental insights and new avenues for controlling resistant microorganisms.

The Research Topic collected 14 papers and for the purpose of this Editorial, they were grouped into three core themes as follows: Thematic group 1—Molecular epidemiology, resistomes, and environmental reservoirs; Thematic group 2—Social dynamics, quorum sensing, and transcriptional regulation and Thematic group 3—Innovative therapeutic interventions designed to bypass classical resistance pressures.

## Thematic group 1—Molecular epidemiology, resistomes, and environmental reservoirs

This group of articles brings studies that map the genomic evolution, and transmission of antimicrobial resistance across diverse clinical, environmental, and wildlife matrices. These articles set the broader ecological and global “One Health” context of how resistance persists and disseminates in natural ecosystems, clinical settings, or in human-built environments.

The work by Shi et al. used metagenomic sequencing to characterize the gut microbiomes of two non-migratory bird species, *Pseudopodoces humilis* and *Pyrgilauda ruficollis*, inhabiting the extreme environment of the Qinghai–Tibet Plateau. The authors identified convergent patterns of microbiome adaptation, including enrichment of pathways related to carbohydrate metabolism and genetic information processing, potentially reflecting adaptation to high-altitude conditions. Notably, despite the relatively low abundance of some resistance-associated taxa, the birds harbored a pervasive core resistome comprising 153 antibiotic resistance ontologies (AROs) spanning major clinical antibiotic classes. The study highlights the potential role of wildlife as reservoirs of antimicrobial resistance.

Moving to the clinical setting, the study by Li et al. investigated the molecular epidemiology and genomic evolution of 123 clinical carbapenem-resistant *Enterobacter cloacae* complex (CRECC) isolates collected at a medical center in Northeast China between July 2019 and March 2025. *Enterobacter hormaechei* accounted for 92.7% of the isolates, with ST1120 predominating during the early study period, followed by a marked shift toward the high-risk ST171 lineage. The resistance landscape was dominated by metallo-β-lactamase genes, particularly *bla*NDM-5 (55.3%) and *bla*NDM-1 (39.0%), while all isolates showed high-level resistance to meropenem and imipenem. Whole-genome analysis further revealed that IncX3 was the predominant plasmid replicon (69.9%) and was strongly associated with *bla*NDM-5, with all 68 *bla*NDM-5-positive isolates carrying IncX3 plasmids. Core-genome SNP analysis identified highly related isolates, providing evidence of recent clonal transmission. Conjugation experiments confirmed horizontal transfer of *bla*NDM-1- and *bla*NDM-5-carrying plasmids in several isolates, with acquisition of these plasmids conferring markedly increased carbapenem MICs in the recipient strain. These findings indicate that the regional dissemination of CRECC is driven by both the expansion of high-risk *E. hormaechei* clones and the horizontal spread of *bla*NDM through IncX3 plasmids, highlighting the combined importance of clonal transmission and mobile resistance determinants in the persistence of carbapenem resistance.

Still on the AMR at the clinical setting, Xiong et al. investigated six clinical isolates of the emerging multidrug-resistant pathogen *Elizabethkingia anophelis* recovered from hospitalized patients in Shanghai. This nosocomial pathogen is usually transmitted by contaminated medical devices causing infections like bacteremia, meningitis and pneumonia that are usually overlooked by conventional diagnostics in addition to being hard to treat. Using hybrid Illumina/PacBio WGS in combination with antimicrobial susceptibility testing and comparative genomics, the authors identified extensive resistance to β-lactams, fluoroquinolones, carbapenems, and aminoglycosides, reflecting five major resistance mechanisms: antibiotic efflux, target alteration, antibiotic inactivation, target replacement, and reduced membrane permeability. Phylogenetic analysis separated the isolates into two distinct clusters, with one group showing closer relationships to East and Southeast Asian strains and the other displaying affinities with isolates from geographically diverse origins, suggesting phylogenetic divergence and potentially independent introduction events. The isolates also carried a diverse repertoire of virulence determinants. Notably, despite their extensive drug resistance, all six isolates remained susceptible to minocycline (MIC ≤ 1 μg/mL), indicating a potential therapeutic option. The particularly distinctive genomic profile of strain EA5, including differences in resistance- and virulence-associated genes, highlights the heterogeneity of *E. anophelis* and the critical role of genome-level characterization in surveillance and clinical practice.

Moving on to a work that compared completely different scenarios, Krämer et al. examined whether the distinct selective pressures of everyday and extreme human-built environments shape bacterial resistance by comparing isolates recovered from high-touch surfaces in German schools and on the International Space Station (ISS). As part of the “Touching Surfaces” experiment, the authors characterized *Staphylococcus* spp. and *Micrococcus luteus* isolates for antibiotic susceptibility and tolerance to spaceflight-relevant stressors, including prolonged desiccation, X-ray irradiation, and hydrogen peroxide exposure. Contrary to the expectation that the ISS environment might select for enhanced resistance, space-derived isolates did not show an overall increase in antibiotic resistance, desiccation tolerance, or X-ray resistance compared with terrestrial isolates. Both ISS and school isolates of *Staphylococcus* showed increased survival following hydrogen peroxide treatment relative to their corresponding type strains, while isolates of *M. luteus* from both environments exhibited enhanced early-stage biofilm formation. Notably, *S. epidermidis* from the school environment also showed increased biofilm formation, highlighting that everyday human-built environments can select for traits relevant to persistence and biofilm-associated infections. Overall, the findings suggest that extreme environments such as the ISS do not necessarily drive broad increases in bacterial resistance, but that human-built environments can select for persistence-associated traits, emphasizing the importance of habitat-specific surveillance of microbial adaptation.

## Thematic group 2—Social dynamics, quorum sensing, and transcriptional regulation

This group of articles focuses on the biochemical, genetic, and social networks that govern how bacteria communicate, coordinate stress responses/gene expression, and share protective resources. These studies span AMR at the community level, including the role of “public goods” which are shared by producing and non-producing organisms, the effect of sub-inhibitory concentrations of antibiotics over time on QS-regulated phenotypes and adaptation, as well as the description of a previously underexplored QS system in *Rhodococcus erythropolis* and an uncharacterized MarR-family transcriptional regulator in *Mycobacterium tuberculosis*.

The work by Wang et al. is a review of the social interactions and AMR at the community level. It addresses cooperative-antibiotic resistance (CoopAR), beyond biofilms, as a community-level phenomenon in which resistant bacteria protect susceptible or less-resistant neighbors by modifying the local environment or bacterial physiology. The authors examine a broader range of extracellular “public goods,” including antibiotic-degrading enzymes such as β-lactamases, chloramphenicol acetyltransferase, outer membrane vesicles, metabolites, and signaling molecules, which can reduce local antibiotic concentrations and create protective microenvironments. Importantly, these benefits can extend beyond the producing cells, allowing susceptible “cheaters” to survive antibiotic exposure without bearing the metabolic cost of producing the protective factors, thereby generating complex social and eco-evolutionary dynamics. The work also emphasizes that many of these mechanisms can operate in planktonic and polymicrobial communities and that the genes encoding extracellular resistance factors may undergo horizontal transfer, potentially spreading cooperative resistance across species. The authors argue that antibiotic resistance should be considered not only as individuals' genetic traits but also as a collective property of microbial communities, with important implications for the design of antimicrobial strategies that target or disrupt these cooperative interactions.

By investigating how sub-inhibitory meropenem exposure drives adaptive changes in *Acinetobacter baumannii*, Jiang et al. showed how these responses are regulated by QS. The authors used a Δ*abaI* mutant of *A. baumannii*, which is deficient in producing acyl-homoserine lactone (AHL) QS-signaling molecules. The *abaI* encodes the autoinducer synthase AbaI, a homolog of the classical LuxI-synthase found in LuxI/LuxR QS-systems in Gram-negative bacteria. Using the wild-type strain and an isogenic Δ*abaI* mutant, together with a 7-day meropenem evolution experiment, the authors showed that sub-MIC antibiotic pressure increased the meropenem MIC of the wild-type strain from 1 to 8 μg/mL, whereas the QS-deficient mutant showed a smaller increase (0.5 to 1 μg/mL). Meropenem exposure also enhanced surface motility, biofilm formation, adhesion to and invasion of A549 epithelial cells, and virulence in *Galleria mellonella* in the wild-type, whereas most of these adaptive responses were substantially impaired following *abaI* deletion. Complementation or exogenous addition of AHLs restored several of these phenotypes. Transcriptomic analysis further revealed extensive transcriptional remodeling under antibiotic stress, with enrichment of pathways related to QS, biofilm formation, ABC transporters, two-component systems, and β-lactam resistance. Network analysis identified *abaI, abaR*, and *bap* among key genes associated with resistance and virulence. Intriguingly, biofilm formation remained enhanced in the Δ*abaI* mutant, suggesting that alternative regulatory pathways can compensate for the loss of QS in this phenotype. Overall, the study demonstrates that sub-inhibitory antibiotic exposure can activate QS-dependent regulatory networks that couple antimicrobial resistance with motility, biofilm development, host interaction, and virulence, highlighting QS as a potential target for limiting antibiotic-driven adaptation in *A. baumannii*.

Also on the QS research front, Bizière-Maco et al. characterized a previously underexplored QS system based on γ-butyrolactones (GBLs) in *R. erythropolis* R138, expanding a signaling mechanism traditionally associated with *Streptomyces* to another important genus of *Actinomycetota*. Comparative genomic analyses revealed that *Rhodococcus* species broadly conserve *scbA* and *scbR* homologs within a highly conserved GBL gene cluster, with additional genes encoding a putative LuxR-like transcriptional regulator and a FAD-dependent monooxygenase. In *R. erythropolis* R138, structural modeling supported the functional conservation of ScbA and ScbR, while LC-MS and a GBL-specific reporter assay demonstrated the production of biologically active GBL-like molecules. Deletion of *scbA* abolished production of these signals and eliminated the ability of *R. erythropolis* to induce sporulation and colony development in a *S. coelicolor* reporter strain, providing functional evidence for GBL-mediated interspecies communication. Transcriptional analyses further indicated that both ScbR and a previously unexpected LuxR-like regulator contribute to the regulation of *scbA* expression, suggesting a complex regulatory architecture. Together, the findings demonstrate that functional GBL-based QS is conserved and active in *R. erythropolis* and potentially widespread across the genus, while also revealing interspecies signaling between *Rhodococcus* and *Streptomyces*.

Finaly, on the intracellular signaling network, Abudukadier et al. investigated the previously uncharacterized MarR-family transcriptional regulator Rv0737 in *M. tuberculosis*, together with its homolog Ms_1492 in the model organism *Mycobacterium smegmatis*. Overexpression of Rv0737 markedly impaired bacterial growth, cell division, and biofilm formation and increased susceptibility to oxidative stress and to the cell wall-targeting antibiotic isoniazid, whereas deletion of *ms_1492* produced changes in cell-envelope permeability, lipid composition, and ATP synthesis and conferred mild tolerance to H_2_O_2_ and isoniazid. Omics analyses revealed extensive alterations in lipid metabolism, including changes in multiple classes of polar lipids, while electrophoretic mobility shift assays demonstrated that Rv0737 and Ms_1492 directly bind the promoter of the *sigL-rslA* operon, which encodes an alternative sigma factor and its anti-sigma factor. Further expression analyses showed that Rv0737/Ms_1492 positively regulates this operon and influences downstream genes involved in lipid and fatty-acid metabolism. Together, the findings establish a direct regulatory connection between a MarR-family transcription factor and the SigL-regulatory pathway, linking transcriptional control to cell-envelope lipid remodeling, membrane permeability, oxidative-stress responses, bacterial growth, and isoniazid tolerance. The study therefore illustrates how intracellular regulatory networks can coordinate multiple physiological traits relevant to antimicrobial adaptation and identifies the Rv0737–SigL axis as a potential target for strategies against drug-resistant tuberculosis.

## Thematic group 3—Innovative therapeutic interventions designed to bypass classical resistance pressures

This final translationally focused group of articles highlights innovative, target-specific approaches designed to disable biofilms, harness molecular synergies, and exploit bacterial physiological vulnerabilities.

Kiran et al. investigated whether QS-regulated metabolites produced by *Pseudomonas aeruginosa* can potentiate conventional antibiotics against multidrug-resistant bacteria. Using metabolites obtained from *P. aeruginosa* strain MC9 and identified by mass-spectrometry as pyocyanin, rhamnolipids, 4-hydroxy-2heptylquinoline (PQS), and its precursor 2-heptyl-3-hydroxy-4(1H)-quinolone (HHQ), the authors evaluated their individual antimicrobial activity and their ability to enhance the activity of conventional antibiotics against clinical isolates of *Salmonella* Typhimurium, *Salmonella* Typhi, and *Escherichia coli*. Although PQS and HHQ showed no direct inhibitory activity against the tested Enterobacteriaceae at concentrations up to 5 mg/mL, all four QS-associated metabolites enhanced the activity of selected antibiotics at much lower concentrations. Pyocyanin produced the strongest effect, increasing the inhibition zone by 300% when combined with sulfamethoxazole/trimethoprim against *S*. Typhimurium, while HHQ increased the inhibition zone by 257% when combined with ampicillin against *E. coli*. Rhamnolipids and PQS also produced substantial synergistic effects with ceftriaxone and amoxicillin/clavulanate, respectively, and growth-curve experiments confirmed dose-dependent suppression of bacterial growth for the most effective combinations. These findings reveal that *P. aeruginosa* QS metabolites can have biological activities extending beyond intercellular signaling and may function as antibiotic-potentiating agents, providing a striking example of how microbial signaling molecules can be repurposed to enhance conventional antimicrobial therapy against resistant bacteria. However, as the authors emphasize, the underlying mechanisms and the safety of using such metabolites require further investigation before therapeutic application.

Tareau et al. studied the anti-biofilm potential of Stigmurin, an antimicrobial peptide originally identified in the venom of the scorpion *Tityus stigmurus*, together with six lysine-enriched synthetic analogs designed to modify the peptide's physicochemical and structural properties. Although neither Stigmurin nor its analogs significantly inhibited the growth of *P. aeruginosa* or prevented biofilm formation under the conditions tested, several peptides were able to disrupt pre-established biofilms at concentrations that did not affect bacterial viability, indicating a predominantly non-bactericidal mechanism. In static assays, Stigmurin reduced mature biofilm biomass by approximately 47% after 24 h, while StigA8 and StigA16 achieved reductions of approximately 39% and 38%, respectively, at 10 μM. StigA31 analog retained substantial activity at nanomolar concentrations. In agreement with the biofilm assays, microscopy studies showed marked reductions in biofilm biovolume and average thickness after treatment under both static and dynamic flow conditions. Structural analyses by NMR revealed that StigA31 adopts an amphipathic α-helical conformation, and its activity was associated with changes in the biofilm matrix rather than stimulation of pyocyanin production. Stigmurin itself altered bacterial membrane fluidity, whereas StigA31 appeared to act through a distinct mechanism that may be related to its peptide conformation. The study demonstrates that rational modification of a natural venom-derived peptide can generate compounds capable of disrupting mature biofilms without exerting strong bactericidal pressure, highlighting peptide-based biofilm dispersal as a promising strategy for addressing persistent infections and complementing conventional antimicrobial approaches.

Keshavarz et al. evaluate two synthetic derivatives of the human cathelicidin LL37, FK13a1-NH_2_ and KR12-NH_2_, against three periodontal bacteria, *Fusobacterium nucleatum, Porphyromonas gingivalis*, and *Streptococcus oralis*, under conditions that mimic cigarette smoke exposure. Both peptides inhibited planktonic *P. gingivalis* and *F. nucleatum*, with KR12-NH_2_ showing particularly strong activity against *P. gingivalis*: the combination of 500 μg/mL KR12-NH_2_ with 50% cigarette smoke extract (CSE) produced 98% inhibition of *P. gingivalis* growth, indicating that tobacco exposure did not compromise its antimicrobial activity. Checkerboard experiments showed that the interaction between KR12-NH_2_ and CSE was pronounced against *P. gingivalis*, whereas the effects against the other species were more variable. In addition, both peptides significantly reduced the thickness of established single-species biofilms, although their effects were considerably less pronounced in complex multispecies biofilms. Overall, the study demonstrates that LL37-derived peptides can retain substantial antimicrobial and anti-biofilm activity under tobacco-associated conditions, suggesting that environmental conditions can unexpectedly modulate the efficacy of antimicrobial peptides. These findings support the potential of synthetic LL37 derivatives as alternatives or complement to conventional antibiotics in periodontal infections, particularly in the clinically relevant context of smoking.

Wickham et al. investigate whether the transient newly released (NRel) phenotype, previously observed after reagent-mediated biofilm disruption, can also be induced through rapid physical disruption. Across four clinically relevant pathogens, non-typeable *Haemophilus influenzae* (NTHI), methicillin-resistant *Staphylococcus aureus* (MRSA), *Pseudomonas aeruginosa*, and *Streptococcus pneumoniae*, the authors show that rapid mechanical disruption, including pipetting, sonication, scraping, and vortexing, releases biofilm-resident cells that become markedly more susceptible to antibiotics than their planktonic counterparts. The effect was also reproduced in NTHI using a cationic-depletion approach, demonstrating that NRel induction is not dependent on a particular chemical reagent. NRel phenotype was characterized by three coordinated features: enhanced antibiotic susceptibility, a transient time-dependent response, and a distinctive gene-expression profile. In NTHI, the authors further show that antibiotic sensitivity changes rapidly after release, consistent with a physiological transition rather than a stable genetic alteration, while qRT-PCR confirmed differential expression of previously identified NRel-associated genes. The authors propose that the rate of biofilm disruption is the critical determinant, because cells released abruptly from the protective biofilm environment may not have sufficient time to activate the physiological programs required for survival in the planktonic state. Thus, rather than simply removing the physical barrier of the biofilm, rapid disruption appears to temporarily place bacterial cells in a vulnerable physiological state that can be exploited therapeutically, providing a potential strategy for converting biofilm-associated antibiotic tolerance into antibiotic susceptibility.

Regarding the use of bioactive compound combinations, Chen et al. investigated the synergistic antibiofilm activity of sodium fluoride (NaF) and the green tea polyphenol epigallocatechin gallate (EGCG) against *Streptococcus mutans*, integrating omics analyses to elucidate the mechanisms underlying their combined effects. Checkerboard assays identified an effective low-dose combination of 62.5 ppm fluoride and 0.5 mg/mL EGCG, which produced more than 90% inhibition of biofilm formation with a fractional inhibitory concentration (FIC) index of 0.5, indicating synergy. The combination markedly reduced biofilm biomass and viable cells while disrupting microcolony organization and decreasing water-insoluble extracellular polysaccharide (EPS) production, although the reduction in EPS was not always proportional to the reduction in total biofilm biomass, suggesting additional mechanisms beyond matrix disruption. Multi-omics analysis revealed a concentration-dependent reorganization of bacterial physiology, with low-dose treatment particularly affecting redox-associated processes and purine metabolism, while higher-dose treatment produced broader changes involving EPS synthesis, amino acid and carbohydrate metabolism, and stress-response pathways. The integrated data therefore indicate that NaF and EGCG do not simply exert additive antimicrobial effects but act on multiple interconnected processes governing biofilm development, including extracellular matrix production, redox homeostasis, nucleotide metabolism, and central cellular energy metabolism. The study provides a mechanistic basis for combining fluoride with EGCG and suggests that carefully optimized low-dose combinations could enhance the antibiofilm effects of conventional fluoride-based strategies for caries prevention.

Finally, Kamphof et al. investigated whether temperatures within the physiological range can be exploited as a non-pharmacological strategy to reduce bacterial persistence and improve antibiotic prophylaxis against prosthetic-joint infections (PJI). Using clinical isolates of methicillin-sensitive *S. aureus*, methicillin-sensitive *Staphylococcus epidermidis*, and *Escherichia coli*, the authors compared bacterial survival, surface adhesion, and antibiotic susceptibility after 24 h exposure to 32, 37, 40, and 42 °C, with or without cefazolin or gentamicin. Increasing the temperature from 32 to 42 °C substantially enhanced the susceptibility of *S. aureus* to both antibiotics, reducing the minimum concentration required for eradication from 0.5 to 0.05 μg/mL, while almost complete eradication of *S. aureus* was achieved at 42 °C even in the absence of antibiotics. *S. epidermidis* also became markedly more susceptible to cefazolin at elevated temperatures, with 0.1 μg/mL cefazolin producing a 6-log reduction in bacterial numbers at 42 °C, whereas antibiotic efficacy was impaired under the hypothermic condition of 32 °C. In contrast, *E. coli* showed little response to temperature changes, indicating that the effect was strongly species dependent. They suggest that thermal stress may damage membrane proteins and reduce respiratory capacity and membrane polarization, thereby increasing bacterial vulnerability to antibiotics whose mechanisms prevent repair of cell-wall or protein damage. Their findings indicate that maintaining or mildly increasing tissue temperature within the physiological range may enhance antibiotic-mediated killing of the Gram-positive pathogens commonly associated with PJI, providing a potentially simple adjunctive approach to infection prevention in orthopedic surgery. The clinical feasibility and safety of the deliberate application of elevated temperatures requires further investigation.

## Conclusion

Taken together, the 14 contributions in this Research Topic illustrate that AMR is not solely a property of individual bacterial cells, but a dynamic phenomenon shaped by microbial interactions, regulatory networks, ecological conditions, and collective behavior. The studies span diverse settings that go from clinical pathogens and healthcare environments to wildlife, human-built environments, extreme habitats, and polymicrobial communities, demonstrating how resistance can emerge and disseminate through clonal expansion, mobile genetic elements, environmental adaptation, and cooperative protection. At the mechanistic level, the contributions highlight the importance of intracellular regulatory pathways and intercellular communication, including QS and social cooperation, in coordinating resistance with biofilm formation, virulence, metabolism, and stress adaptation. Several studies moved beyond characterization toward exploiting microbial vulnerabilities, demonstrating that disrupting biofilms, targeting signaling pathways, combining natural compounds with conventional antimicrobials, using antimicrobial peptide derivatives, or even manipulating physiological conditions can increase bacterial susceptibility without necessarily relying on higher antibiotic pressure. These studies reinforce the concept that understanding how microorganisms communicate, adapt, and respond cooperatively can open new avenues for combating AMR. This Research Topic highlights the importance of targeting the broader microbial systems that sustain resistance, providing fundamental insights into microbial ecology and pointing toward new directions for developing innovative antimicrobial and antivirulence strategies.
